# The complete chloroplast genome of *Litsea molis* Hemsl. (Lauraceae): genome structure and phylogenetic analysis

**DOI:** 10.1080/23802359.2020.1790315

**Published:** 2021-01-21

**Authors:** Xin-yu Wang, Huan Hu, Dan Zhang

**Affiliations:** a Key Laboratory of Medical Electrophysiology, Ministry of Education, Institute of Cardiovascular Research, Southwest Medical University, Luzhou, P. R. China; b Institute of Life Sciences, Zunyi Medical University, Zunyi, P. R. China

**Keywords:** *Litsea molis* Hemsl, chloroplast genome, oil species, medicine plant, phylogenetic analysis

## Abstract

*Litsea molis* Hemsl. is a precious wild aromatic oil tree species and important medicinal plant. In this study, we sequenced and characterized the chloroplast genome of *L. molis* for the first time. Its complete chloroplast genome is 152,311 bp in length, containing a large single copy region of 93,258 bp and a small single copy region of 18,917 bp separated by a pair of inverted repeat regions of 20,068 bp. The chloroplast genome contains 130 unique genes, including 85 protein-coding genes, 37 tRNA and 8 rRNA genes. Phylogenetic analysis based on chloroplast genome sequences of 36 plants from the family Lauraceae showed that *L. molis* is more closely related to species of *Lindera* genus than other genera in Lauraceae.

*Litsea mollis* Hemsl., a traditional edible spice and medicinal plant with a long history in China, is a deciduous shrubs or small trees in the *Litsea* genus of Lauraceae. The seeds contain ca. 25% oil and are used as a main ingredient in soaps. The fruit is processed for its aromatic oil (3%–5%) (Li et al. 1982). Ingredients from volatile oil of *L. molis* can produce natural pesticides, grain and clothing anthelmintic storage agents and natural preservatives. Volatile oil in both fruit and leaf have obvious antibacterial and antifungal activity. In terms of medicinal value, the roots and fruit are used medicinally (Lin et al. 2000; Cai et al. 2019). A variety of biological activities in *L. molis* have beneficial effective on human health, such as roots and fruits can cure gas pain, strain injury, diarrhea, analgesia, schistosomiasis, allergy and disease of cardiovascular system (coronary heart disease, angina pectoris) and so on (Yang et al. 2020). However, there are very few studies on the *L. molis*, which greatly limit the development and utilization of *L. molis*. Therefore, we assembled and analyzed the complete chloroplast genome of *L. molis* for the first time, which can provide insight and evidence for its genetic background and evolution.

Fresh leaves of *L. mollis* were collected from Longquan Mountain (coordinates: CHN, 26°11'41.26′'N, 107°49'10.32E''), Danzhai County, Guizhou province, China. The specimen was stored at College of life sciences, Sichuan University (specimen code LLM20001). Total genomic DNA were extracted with a modified CTAB method (Doyle and Doyle 1987) and sequencing was carried out by the Illumina pair-end technology to obtain bout 5.4 Gbp of high-quality reads of *L. mollis* on the Hiseq2000 platform. Firstly, we obtained 10 million high quality pair-end reads using Trimmomatic v0.35 (Bolger et al. 2014) and custom Perl script for *L. mollis*. With the chloroplast genome of all published Lauraceae chloroplast genomes as the reference sequences, we assembled the complete chloroplast genome from the clean reads by the GetOrganelle pipe-line (Jin et al. 2018). Secondly, we annotated the plastid genomes using PGA (Qu et al. 2019) and corrected the annotation with Geneious v11.1.5.0 (Kearse et al. 2012) and Sequin v13.70 (http://www.ncbi.nlm.nih.gov/Sequin/). The gene map of the chloroplast genome was generated using OGDRAW v1.3 (Greiner et al. 2019). Finally, a complete chloroplast genome of *L. mollis* was obtained and submitted to Genbank (accession number MT472628).

The complete chloroplast genome of *L. mollis* is 152,311 bp in length, containing a pair of inverted repeat (IR) regions of 20,068 bp, a large single copy region of 93,258 bp, and a small single copy region of 18,917 bp. The chloroplast genome of *L. mollis* contains 130 unique genes, including 85 protein-coding genes, 37 tRNA genes, and 8 rRNA genes (4 rRNA species). Among these genes, 94 of them exist as single copy, but 4 protein-coding genes (i.e. ndhB, rps7, ycf12, and ycf68), 6 tRNA genes (i.e. trnA-UGC, trnI-GAU, trnL-CAA, trnN-GUU, trnR-ACG, trnV-GAC), and 4 rRNA genes (i.e. 4.5S, 5S, 16S and 23S rRNA) present in double copies. In general, the overall GC content of the whole chloroplast genome is 39.2%, whereas the corresponding values of the LSC, SSC, and IR regions are 37.95%, 33.95%, and 44.40%, respectively.

To further investigate its phylogenetic position, Cupressus gigantea was used as the outgroup, the phylogenetic analysis of 36 plastid genomes from published species of Lauraceae were analyzed using MEGA7.0 (with 1,000 bootstrap replicates) (Kumar et al. 2016). The result indicated that *L. molis* is more closely related to species of *Lindera* genus than other genera in Lauraceae (Figure 1).

**Figure 1.**
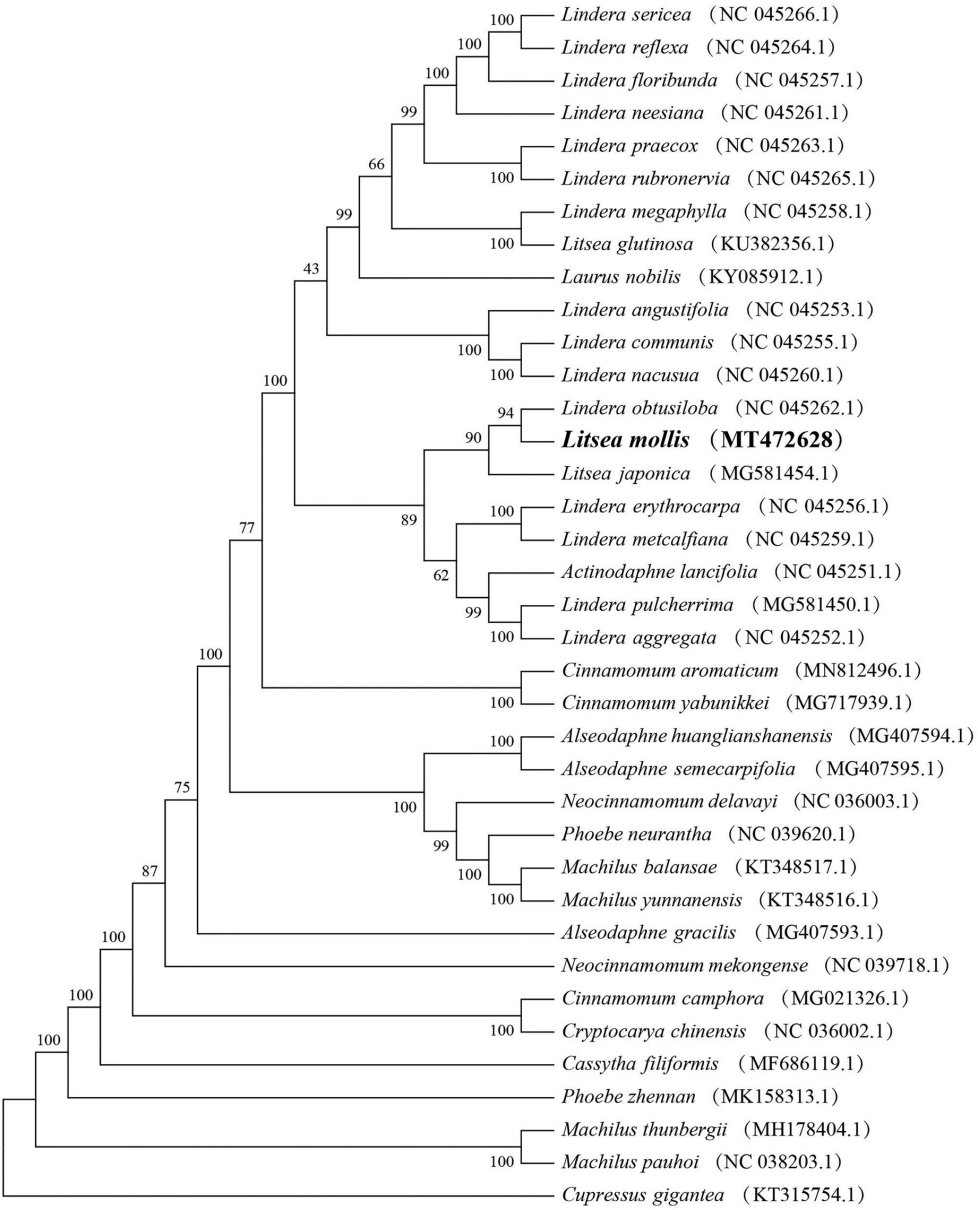
Phylogenetic relationships based on complete chloroplast genome sequences from L. molis and other members of from Lauraceae (All the sequences were downloaded from NCBI GenBank).

## Data Availability

The data that support the findings of this study is openly available in Genbank at [https://www.ncbi.nlm.nih.gov/genbank/], reference number MT472628.
